# The impact and causal directions for the associations between diagnosis of ADHD, socioeconomic status, and intelligence by use of a bi-directional two-sample Mendelian randomization design

**DOI:** 10.1186/s12916-022-02314-3

**Published:** 2022-04-11

**Authors:** Madeleine Michaëlsson, Shuai Yuan, Håkan Melhus, John A. Baron, Liisa Byberg, Susanna C. Larsson, Karl Michaëlsson

**Affiliations:** 1 Department of Education, Dalarna University, Falun, Sweden; 2 Unit of Cardiovascular and Nutritional Epidemiology, Institute of Environmental Medicine, Karolinska Institutet, Stockholm, Sweden; 3 Department of Medical Sciences, Clinical Pharmacology, Uppsala University, Uppsala, Sweden; 4 Department of Medicine, University of North Carolina School of Medicine, Chapel Hill, NC USA; 5 Department of Epidemiology, Gillings School of Global Public Health, University of North Carolina, Chapel Hill, NC USA; 6 Department of Surgical Sciences, Unit of Medical Epidemiology, Uppsala University, Uppsala, Sweden

**Keywords:** Attention-deficit/hyperactivity disorder, ADHD, Socioeconomic status, Education, Intelligence, Income, Townsend deprivation index, Mendelian randomization, Gene, GWAS

## Abstract

**Background:**

Previous studies have reported associations between attention-deficit/hyperactivity disorder (ADHD) and lower socioeconomic status and intelligence. We aimed to evaluate the causal directions and strengths for these associations by use of a bi-directional two-sample Mendelian randomization (MR) design.

**Methods:**

We used summary-level data from the largest available genome-wide association studies (GWAS) to identify genetic instruments for ADHD, intelligence, and markers of socioeconomic status including the Townsend deprivation index, household income, and educational attainment. Effect estimates from individual genetic variants were combined using inverse-variance weighted regression.

**Results:**

A genetically predicted one standard deviation (SD) increment in the Townsend deprivation index conferred an odds ratio (OR) of 5.29 (95% confidence interval (CI) 1.89–14.76) for an ADHD diagnosis (*p*<0.001). A genetically predicted one SD higher education level conferred an OR of 0.30 (95% CI 0.25–0.37) (*p*<0.001), and a genetically predicted one SD higher family income provided an OR of 0.35 (95% CI 0.25–0.49; *p*<0.001). The associations remained after adjustment for intelligence whereas the lower odds of an ADHD diagnosis with higher intelligence did not persist after adjustment for liability to greater educational attainment (adjusted OR 1.03, 95% CI 0.68–1.56; *p*=0.87). The MR analysis of the effect of ADHD on socioeconomic markers found that genetic liability to ADHD was statistically associated with each of them (*p*<0.001) but not intelligence. However, the average change in the socioeconomic markers per doubling of the prevalence of ADHD corresponded only to 0.05–0.06 SD changes.

**Conclusions:**

Our results indicate that an ADHD diagnosis may be a direct and strong intelligence-independent consequence of socioeconomic related factors, whereas ADHD appears to lead only to modestly lowered socioeconomic status. Low intelligence seems not to be a major independent cause or consequence of ADHD.

**Supplementary Information:**

The online version contains supplementary material available at 10.1186/s12916-022-02314-3.

## Background

The diagnosis of attention-deficit/hyperactivity disorder (ADHD) has become highly prevalent during the last two decades [1], but there are considerable regional differences, both within and between countries, in the proportion of children and adolescents with this diagnosis [2–4]. These differences may be explained by true variation in the prevalence or to be attributable to cultural contexts, diagnostic resources, and practice, as well as to biological factors like genetics [5, 6]. Hitherto, there is no established clinically valid biological marker for the neuropsychiatric diagnosis of ADHD [4].

Genetics clearly contribute to ADHD. Family studies indicate a five-fold higher risk in first-degree relatives of cases, and genetic liability to an ADHD diagnosis in children and adolescents is estimated to be 70–80% [7, 8]. Boys have a higher ADHD prevalence than girls [1, 6], but the heritability is higher for girls [8]. To date, genome-wide association studies have indicated single-nucleotide polymorphism (SNP) heritability, the proportion of phenotypic variance explained by all measured and statistically significant SNPs, to be 20%, leaving considerable missing heritability, i.e., the difference between SNP versus twin heritability [9]. This is not uncommon in genome-wide association study (GWAS) research and can be explained by rare or weak genetic variants, which are only captured by very large datasets [10]. Another conceivable explanation for the missing heritability can be moderately strong genetic factors not causally related to the ADHD diagnosis per se, but rather associated with the probability of being diagnosed. Two such contributing factors may be socioeconomic status (SES) and intelligence. Different aspects of SES, such as educational attainment and income, typically display twin heritabilities of 40% [11, 12]. The heritability of intelligence increases linearly, from 40% in childhood to 80% in late adulthood but declines to about 60% after age 80 years [13]. The relative importance of SES, including parental income, education, occupation, and marital status [14] as well as intelligence, in relation to the diagnosis of ADHD has long been debated. Several longitudinal cohort studies have reported a close relationship between socioeconomic disadvantage in childhood and a later diagnosis of an attention deficit/hyperactivity disorder (ADHD) [14–18]. Meta-analyses of measures of SES and their association with ADHD indicate that children in families of low SES are on average at doubled risk of receiving an ADHD diagnosis than their peers in high SES families [14]. Specifically, parental financial difficulties, housing conditions, education, occupation, and marital status have been shown to significantly be associated with children’s likelihood to be diagnosed with ADHD [14–18]. An ADHD diagnosis is also associated with relatively poor academic achievement and work performance [18–20] as well as with moderately lower intellectual capacity [21, 22].

Due to a complex web of interrelated associations, the independence and direction of the associations between a diagnosis of ADHD with specific markers of socioeconomic status and intelligence is unclear [14]. The observational nature of most studies of ADHD’s association with SES and intelligence hampers causal inference due to potential biases from residual confounding and reverse causation.

The Mendelian randomization (MR) design can overcome such biases, thereby strengthening causal inference regarding an exposure-outcome association by leveraging genetic variants as instrumental variables for an exposure [23]. In this approach, causality is inferred from associations between genetic instrument proxies for a putative modifiable risk factor and the outcome of interest. Bidirectional MR, with analyses assessing causality in both directions, can help tease apart causal temporal directions of two related variables [24]. In this case, if low SES or lower intelligence leads to a higher risk of an ADHD diagnosis, then genetic variants associated with lower SES or lower intelligence should be related to higher risk for a diagnosis of ADHD. Conversely, if ADHD is causally related to markers of low SES or intelligence, a genetic variant associated with a higher risk for a diagnosis of ADHD should be associated with lower SES or lower estimated intelligence. One recent Mendelian randomization study showed that genetic liability to higher education was associated with a lower risk of an ADHD diagnosis independently of intelligence but an analysis in the reverse direction displayed a modestly strong association [25]. This implies that lower education contributes as a cause and not a clear consequence of ADHD. Other aspects of SES in relation to an ADHD diagnosis have not been investigated by the use of a Mendelian randomization design.

Our aim was therefore to use a bi-directional two-sample MR design to assess the associations between markers of SES or intelligence and an ADHD diagnosis.

## Methods

We used a two-sample MR design in which the genetic instruments for the exposure, and the outcome are extracted from independent GWAS data sources. Our analyses were bidirectional, first assessing the causal effect of the socio-economic variables on ADHD and then investigating the reverse relationship. Each analysis used multiple genetic variants obtained from publicly available GWAS summary data [26]. None of the data sources investigated both ADHD and socioeconomic status or intelligence, and there is no known subject overlap between the two sources of data [9].

### Data sources

Data regarding the Townsend index of deprivation (TID) [27], household income, educational attainment, intelligence, and ADHD were obtained from four sources.

Genetic instruments for the TID (including the components unemployment, lack of car ownership, lack of home ownership, and household overcrowding) was retrieved from the UK Biobank (*n*=462,464; https://gwas.mrcieu.ac.uk/datasets/ukb-b-10011/) using a *z* score with a mean of −1.29 and a standard deviation (SD) of 3.10. A greater TID corresponds to a lower socioeconomic status. The UK Biobank was also used for a GWAS of self-reported household income (*n*=397,751; https://gwas.mrcieu.ac.uk/datasets/ukb-b-7408/), calculated as the average total household income in 2006–2010 before tax reduction.

We obtained genetic instrument data for education level from the Social Science Genetic Association Consortium (*n*=766,345), based on longitudinal assessment of cohort participants measured at an age of at least 30 [28]. Education level was defined as the number of years of education with durations harmonized across studies according to the International Standard Classification of Education categories [28]. The sample-size-weighted mean of years of education year was 16.8 years with a SD of 4.2 years.

We used Savage et al.’s GWAS meta-analysis of intelligence (children, young adults, middle-aged and older individuals with *n*=269,867) [29]. Included cohorts extracted a single sum score, mean score, or factor score from a multidimensional set of cognitive performance tests in a GWAS, with the exception of the High-Intelligence/Health and Retirement Study in which a logistic regression GWAS was run with “case” status (high intelligence, top 0.03% tail in the normal population) versus controls (a population sample of unselected individuals) [29].

Finally, we included genetic data from the international Psychiatric Genomics Consortium, which involved 20,183 individuals diagnosed with ADHD and 35,191 controls [9]. This GWAS did not include UK Biobank data. There was no heterogeneity of effects when the investigators compared different types of studies [9], including those based on children, those based on adults, and those who used the International Classification of Diseases (ICD) 10 clinical diagnosis of ADHD (code F90.0), ADHD treatment, and those employing continuous quantitative population measures of ADHD-related behaviors [9]. Specifically, in this GWAS [9], the genetic instruments associated with a clinical diagnosis of ADHD and ADHD treatment were also associated with population measures of ADHD-related behaviors. A genetic correlation analysis provided additional evidence that effects were consistent across cohorts included in the analysis [9]. Results based on data collected from populations in Western Europe and North America were accordingly similar. We extracted harmonized GWAS data for all phenotypes through the MR-Base platform [30]. In the current analyses, we restricted our analysis to results based on individuals of the European ancestry.

### Genetic instruments

Genetic instruments, identified at a genome-wide significance threshold of *p*<5×10^-8^, were selected from the corresponding genome-wide association studies. Linkage disequilibrium (defined as *R*^2^>0.01 or clump distance <10,000 kb) between SNPs was assessed based on the 1000 Genomes European reference panel (https://www.internationalgenome.org/). For SNPs in linkage disequilibrium, those with the strongest association with the exposure were retained. Remaining independent SNPs who had met a GWAS-wide significance threshold were used as genetic instruments. SNPs that were unavailable in an outcome dataset were replaced by suitable proxies (minimum linkage disequilibrium *R*^2^=0.8) where available. We removed SNPs without an imputed substitute as well as all palindromic SNPs. Our genetic instruments included 17 SNPs for the TID, 42 SNPs for household income, 219 SNPs for educational attainment, and 132 SNPs for intelligence. These explained 0.13%, 0.53%, 2.8%, and 3.2% of the variance in the TID, household income, educational attainment, and intelligence, respectively. For the analysis with ADHD as the exposure, we used nine (out of 12) [9] conditionally independent SNPs associated with ADHD (Additional file 1: Table S1); these SNPs explained 0.6% of the variance in ADHD.

### Statistical analysis

Since our different phenotypes are linked to each other, we first illustrate these relationships by the genetic correlations of ADHD with attained education, family income, TID, and intelligence using LD score regression [31].

In our main analysis, we assessed the direction and strengths of the associations of ADHD with socioeconomic markers and intelligence in a bi-directional design. We first estimated the impact of our SES variables and intelligence on the odds of ADHD, estimating both the total effect of each variable and the direct effect independent of intelligence and SES, respectively, by use of multivariable Mendelian randomization [32]. Secondly, we examined the association in the reverse direction, i.e., the influence of ADHD on SES and intelligence.

**Fig. 1:**
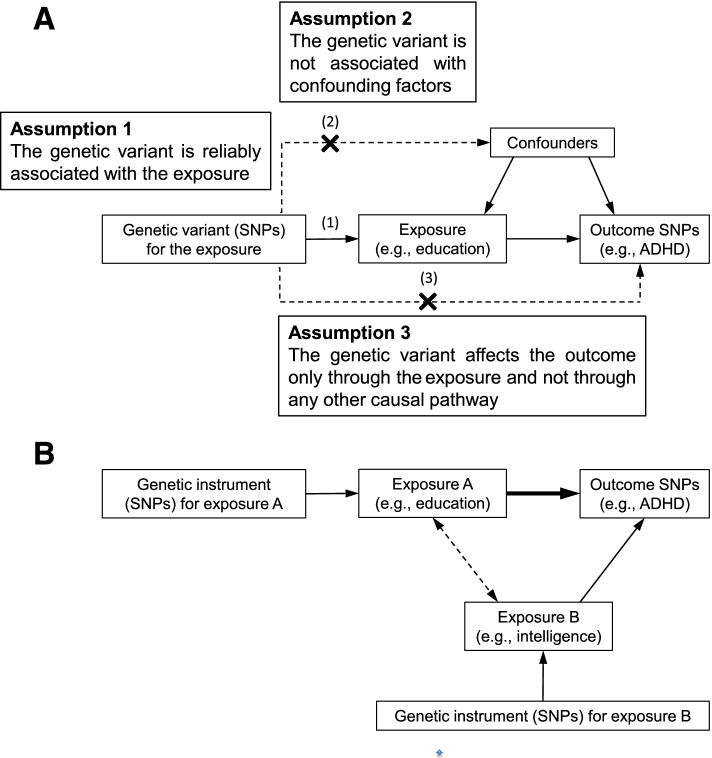
Panel **A** illustrates the assumptions underpinning a Mendelian randomization analysis of the association between an exposure (e.g., education) and an outcome (e.g., ADHD). SNPs indicate single-nucleotide polymorphisms. The arrows represent causal pathways. The dashed arrows represent potential causal associations between variables that would violate the Mendelian assumptions. Panel **B** displays one such possible violation with inclusion of an exposure B (independent exposure, mediator, or confounding factor), in our example proposed to be intelligence. One method to examine the influence of this possible violation is multivariable Mendelian randomization analysis (MVMR) and the inverse-variance weighted method [32] with markers for education adjusted for intelligence. The remaining direct causal effect of education on ADHD is illustrated by the bold arrow

The principal analyses (Fig. 1A) were conducted using an inverse-variance weighted (IVW) approach in a multiplicative random-effects model, which assumes that all SNPs are valid instrumental variables [26, 33] and that the estimates can be interpreted to reflect the total effect of the exposure. In addition, we used multivariable MR analysis and the inverse-variance weighted method [32] with markers for SES adjusted for intelligence, and vice versa (Fig. 1B). This method was used to estimate the independent direct causal effect of each of the exposure on the outcome [32].

We report odds ratios (ORs) of ADHD per one SD increase of genetically predicted socioeconomic marker or intelligence. For ADHD as the exposure, effect sizes are expressed as SD changes in the socioeconomic markers or intelligence per unit increase in genetically predicted log odds ratio of ADHD. A log odds ratio of one thus corresponds to an OR of 2.7, and the average change in socioeconomic markers per doubling of the prevalence of an ADHD diagnosis equals 0.693 (=log_e_ 2) times the causal estimate on the untransformed scale [34]. All statistical tests were two-tailed. Associations that were statistically significant at a *p*-value <0.006 (with Bonferroni correction for 8 main outcomes) were considered to show strong evidence of association. *P*-values <0.05, but higher than 0.006, were regarded as showing suggestive evidence of associations, requiring confirmation.

In sensitivity analyses, the following approaches were applied: (1) the weighted median method, which provides a causal estimate if at least 50% of the weight in the analysis comes from valid instrumental variables [33]; (2) MR-Egger regression, which can detect and adjust for directional pleiotropy but has low precision [33]; (3) the Pleiotropy RESidual Sum and Outlier (MR-PRESSO) method, which can detect and adjust for horizontal pleiotropy by outlier removal [35]; (4) the contamination mixture method, which performs MR robustly and efficiently in the presence of invalid instrumental variables [36]; the (5) MRMix method, which also aims to estimate causal effects in the presence of invalid instruments [37]; the (6) Generalised Summary-data-based Mendelian Randomisation (GSMR) method, which performs MR analysis with multiple near-independent instruments to test for causal associations [38]; and the (7) MRMode, which has been proposed to provide a single causal effect estimate from multiple genetic instruments [39] but, in accordance with MR-Egger, has only moderate precision [37].

With traditional MR methods there remains a concern that valid instrument selection are often violated, leading to false positive findings through correlated horizontal pleiotropy. To avoid this bias, we adopted a new MR method as the eighth (8) sensitivity analysis, using summary effect estimates (MR-CAUSE), to differentiate correlated pleiotropy from causal effects [40]. MR-CAUSE assumes that the relationship between the genetic instrument’s effect on exposure and on outcome is a mixture of both causal and shared correlated pleiotropy. It estimates posterior distributions of a null effect, a shared effect, and a causal effect of the exposure [40, 41], and model fit comparisons are done by Δ Expected Log Pointwise Posterior Density (ELPD).

The TwoSampleMR [30], MendelianRandomization [42], MRPRESSO [35], and MR-CAUSE [40] packages were used for the statistical analyses. We restricted our analysis to participants with the European ancestry.

## Results

Genetic variants with a positive effect on ADHD tended to have a negative association with intelligence and educational attainment: the genetic correlations were −0.37 (95% CI −0.44 to −0.30) and −0.51 (−0.57 to −0.45), respectively. Education and intelligence were genetically related, as expected (*r*=0.73; 95% CI 0.68 to 0.78)), and education was also correlated with household income (*r*=0.77; 95% CI 0.70 to 0.84)) and with TID (*r*=−0.40; 95% CI −0.46 to −0.35). Intelligence was correlated with both household income (*r*=0.64; 95% CI 0.56 to 0.72) and TID (*r*=0.20; 95% CI 0.15 to 0.24).

### Total and direct effect of genetically predicted SES indicators and intelligence on ADHD

**Fig. 2:**
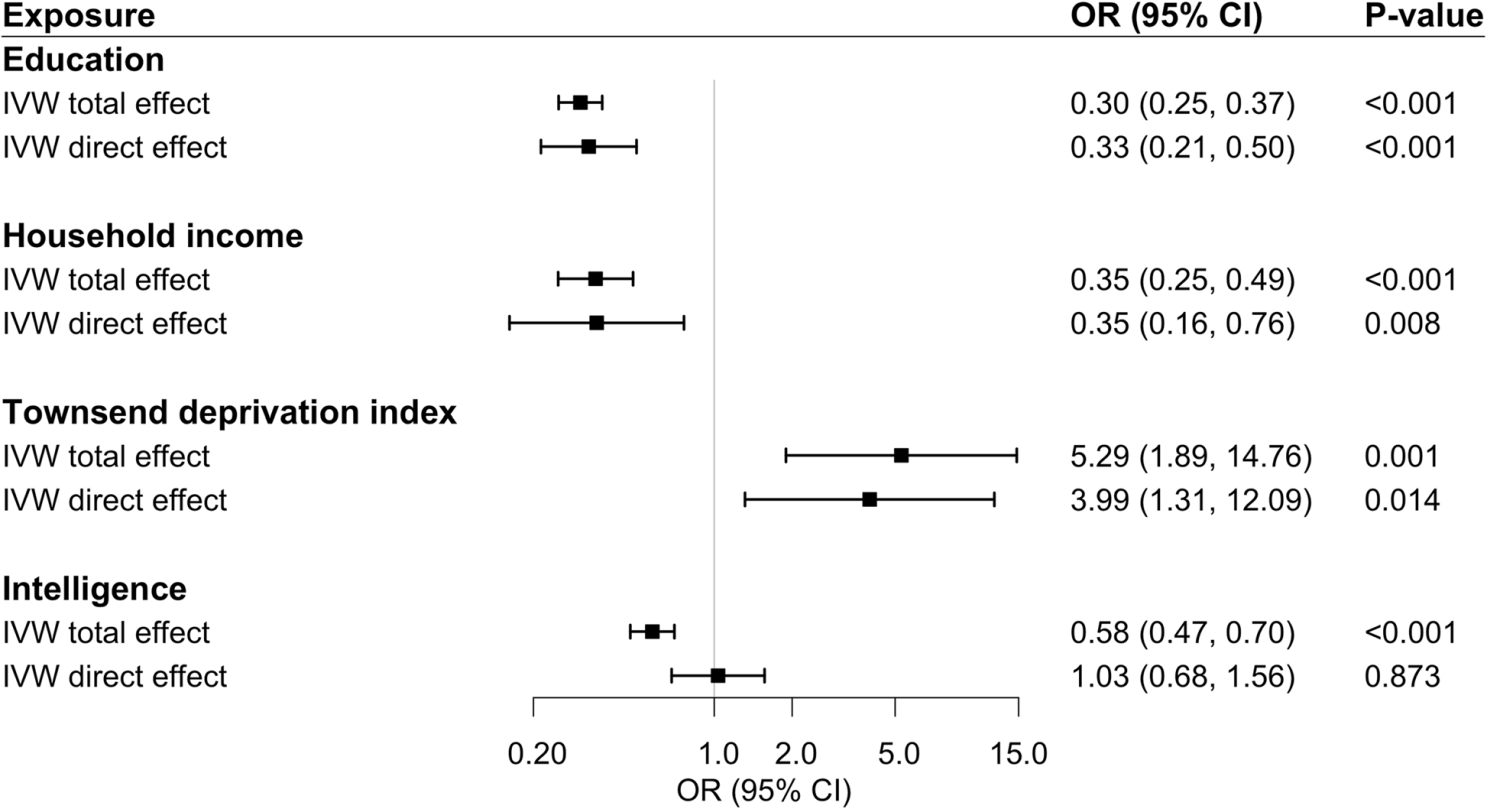
Results of the Mendelian randomization analyses of the odds of ADHD conferred by the liability for one standard deviation increase in attained educational level, household income, Townsend deprivation index, or intelligence. Ordinary IVW estimates are provided for the total effect of the exposure, and MVWR IVW estimates for the direct effect of the exposure. Estimates for SES markers are adjusted for intelligence; that for intelligence is adjusted for education

In IVW-random effect models, genetically predicted TID, household income, and educational attainment were all strongly associated with ADHD (Fig. 2). One SD higher genetically predicted duration of education conferred an OR of 0.30 (95% CI 0.25–0.37) (*p*<0.001), and one SD higher genetically predicted family income gave an OR of 0.35 (95% CI 0.25–0.49; *p*<0.001). A one SD increment in genetically predicated TID conferred an OR of 5.29 (95% confidence interval [CI] 1.89–14.76) (*p*<0.001) for ADHD. The direct effects, after adjustment for intelligence, were similar (Fig. 2), with ORs of 0.33 (95% CI 0.21–0.50), OR 0.35 (95% CI 0.16–0.76), and 3.99 (95% CI 1.31–12.09), respectively.

We also observed (Fig. 2) that intelligence was related to ADHD, with a somewhat more moderate OR of 0.58 (95% CI 0.47–0.70; *p*<0.001) per SD higher predicted intelligence. This lower odds of genetic liability of an ADHD diagnosis with genetically predicted higher intelligence did not remain after adjustment for educational attainment (OR 1.03, 95% CI 0.68–1.56; *p*=0.87). The estimate was also attenuated by the adjustment for household income (OR 0.71, 95% CI 0.50–0.99; *p*=0.04 but not by adjustment for TID (OR 0.61, 95% CI 0.50–0.74; *p*<0.001).

The results were largely the same in sensitivity analyses (Table 1) based on the weighted median, MR-PRESSO methods, and contamination mixture analyses. The MR-Egger analysis showed no clear evidence of horizontal pleiotropy, with the exception of that for household income (*p* = 0.038). The MRMix analysis indicated independent causal effects of education, intelligence, and income (but not TID) on ADHD, while all four exposures remained statistically significant causes of ADHD when data were analyzed by GSMR. We found also similar patterns with MR-Mode although with lower precision of the estimates. Sensitivity analysis by MR-CAUSE (Table 2) showed that intelligence and income, but especially education, remained as independent causes of ADHD, e.g., the causal model of education on ADHD was superior to the shared education model on ADHD.

**Table 1:** Results of the Mendelian randomization sensitivity analyses associating the liability for one standard deviation increase in attained educational level, household income, Townsend deprivation index, and intelligence with the odds of ADHD

Exposure	Method	OR (95% C))	***p***-value
Education (219 SNPs)	Weighted median	0.34 (0.27, 0.43)	<0.001
	MR-Egger	0.39 (0.18, 0.85)	0.018
	MR-PRESSO (3 outliers)	0.30 (0.25, 0.36)	<0.001
	Contamination mixture	0.23 (0.17, 0.32)	<0.001
	MR-Mode	0.58 (0.27, 1.26)	0.168
	MR-Mix	0.37 (0.23, 0.59)	<0.001
	GSMR	0.29 (0.25–0.34)	<0.001
Household income (42 SNPs)	Weighted median	0.49 (0.34, 0.72)	<0.001
	MR-Egger	1.64 (0.38, 7.00)	0.508
	MR-PRESSO (1 outlier)	0.38 (0.29, 0.51)	<0.001
	Contamination mixture	0.37 (0.25, 0.54)	<0.001
	MR-Mode	0.69 (0.34, 1.40)	0.308
	MR-Mix	0.37 (0.23, 0.60)	<0.001
	GSMR	0.28 (0.21, 0.38)	<0.001
Townsend deprivation index (17 SNPs)	Weighted median	5.51 (2.22, 13.71)	<0.001
	MR-Egger	0.04 (0.00, 229.4)	0.482
	MR-PRESSO (2 outliers)	4.86 (2.11, 11.19)	0.002
	Contamination mixture	8.25 (3.78, 27.39)	0.001
	MR-Mode	8.94 (1.58, 50.69)	0.025
	MR-Mix	1.00 (1.00, 1.00)	0.99
	GSMR	4.92 (2.95, 8.21)	<0.001
Intelligence (132 SNPs)	Weighted median	0.59 (0.48, 0.73)	<0.001
	MR-Egger	0.68 (0.27, 1.72)	0.416
	MR-PRESSO (4 outliers)	0.58 (0.48, 0.69)	<0.001
	Contamination mixture	0.53 (0.44, 0.62)	<0.001
	MR-Mode	0.59 (0.37, 0.97)	0.037
	MR-Mix	0.50 (0.31–0.81)	0.005
	GSMR	0.53 (0.46–0.60)	<0.001

**Table 2:** MR-CAUSE analysis associating the liability for one standard deviation increase in attained educational level, household income, Townsend deprivation index, and intelligence with the odds of ADHD

Model 1^a^	Model 2^a^	Δ ELPD^b^	s.e. Δ ELPD	***z***-score	***p***-value
**Education (SNPs=3733)**					
Null	Sharing	−120.0	13	−8.7	2.0e−18
Null	Causal	−120.0	14	−8.6	3.0e−18
Sharing	Causal	−7.3	1.2	−6.2	2.1e−10
**Income (SNPs =73)**					
Null	Sharing	−3.6	1.5	−2.5	0.007
Null	Causal	−9.7	3.4	−2.9	0.0022
Sharing	Causal	−6.1	2.1	−3.0	0.0015
**TDI (SNPs =27)**					
Null	Sharing	−0.31	0.45	−0.70	0.24
Null	Causal	−1.60	2.2	−0.74	0.23
Sharing	Causal	−1.30	1.8	−0.73	0.23
**Intelligence (SNPs=2945)**					
Null	Sharing	−25.0	5.7	−4.4	5.5e−06
Null	Causal	−31.0	7.1	−4.4	5.2e−06
Sharing	Causal	−5.9	1.4	−4.1	1.8e−05

^a^Model 1 and Model 2 refer to the models being compared (null, sharing, or causal)

^b^Model fit is measured by Δ Expected Log Pointwise Posterior Density (ELPD); Negative values indicate that model 2 is a better fit

### Total effect of genetic liability to ADHD on markers of socio-economic status and intelligence

**Fig. 3:**
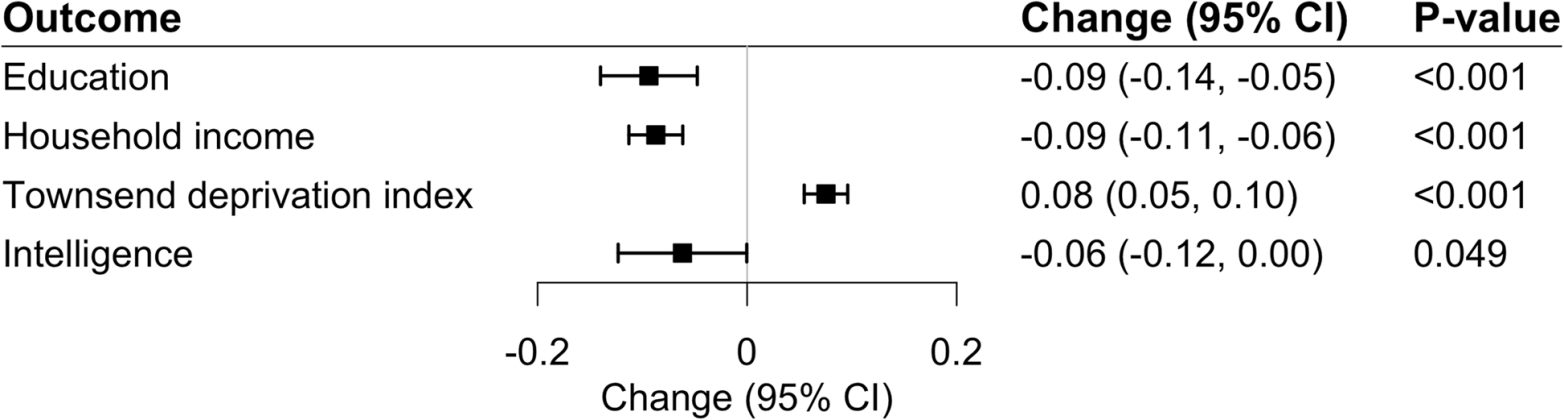
Results of the Mendelian randomization IVW analysis for one standard deviation difference in attained educational level, household income, Townsend deprivation index, or intelligence from genetic liability to ADHD (9 SNPs)

IVW-random effect analysis in the reverse direction indicated that genetic predisposition to ADHD was statistically significantly associated with SES variables but not with intelligence (Fig. 3). However, the strength of the associations in this direction were modest. One log odds ratio of genetically predicted ADHD conferred a 0.09 SD (95% CI 0.05–0.14) lower educational attainment (corresponding to 4.5 months lower education duration), a 0.09 SD (95% CI 0.06–0.11) lower household income, and a 0.08 SD (95% CI 0.05–0.10) higher TID (all *p*<0.001). Expressed in another way, the average change in adverse socioeconomic markers per genetically predicted doubling in the prevalence of the ADHD diagnosis corresponded to only 0.05–0.06 SD units lower SES measures. The modest impact of ADHD on SES and intelligence can theoretically be a consequence of weak instrument bias given that we only included 9 ADHD SNPs in our analyses. However, the F-statistics with values between 181 and 514 do not indicate such bias.

The results were similar in sensitivity analyses based on the weighted median, MR-PRESSO methods, and contamination mixture approach (Table 3). There was limited evidence of unbalanced and correlated horizontal pleiotropy as estimated by MR-Egger regression (*p*-values from 0.11 to 0.78) as also indicated by analyses using MR-Mode, MRMix (Table 3), and MR-CAUSE (Table 4). The number of SNPs for ADHD (*n*=9) precluded an analysis by use of GSMR.

**Table 3:** Results of the Mendelian randomization sensitivity analyses using summary-level data from genetic liability to ADHD (9 SNPs) to a standard deviation change in attained educational level, household income, Townsend deprivation index, and intelligence

Outcome	Method	OR (95% CI)	***p***-value
Education	Weighted median	−0.07 (−0.1, −0.03)	<0.001
	MR-Egger	0.03 (−0.15, 0.21)	0.760
	MR-PRESSO (4 outliers)	−0.06 (−0.10, −0.02)	0.046
	Contamination mixture	−**0.12 (**−**0.19, 0.00)**	**<0.001**
	MR-Mode	−0.08 (−0.15, 0.00)	0.078
	MR-Mix	−0.15 (−0.21, −0.09)	<0.001
	GSMR (<10 SNPs)	NA	
Household income	IVW-random effects	−0.09 (−0.11, −0.06)	<0.001
	Weighted median	−0.09 (−0.12, −0.05)	<0.001
	MR-Egger	−0.03 (−0.14, 0.07)	0.564
	MR-PRESSO (0 outlier)	NA	NA
	Contamination mixture	−0.09 (−0.11, −0.07)	<0.001
	MR-Mode	−0.08 (−0.14, −0.02)	0.026
	MR-Mix	−0.04 (−0.06, −0.02)	0.001
	GSMR (<10 SNPs)	NA	
Townsend deprivation index	Weighted median	0.06 (0.04, 0.09)	<0.001
	MR-Egger	0.01 (−0.07, 0.09)	0.762
	MR-PRESSO (0 outliers)	NA	NA
	Contamination mixture	0.08 (0.05, 0.09)	<0.001
	MR-Mode	0.07 (0.03, 0.10)	0.003
	MR-Mix	0.07 (−0.02, 0.16)	0.132
	GSMR (<10 SNPs)	NA	
Intelligence	Weighted median	−0.05 (−0.10, −0.01)	0.030
	MR-Egger	−0.24 (−0.49, 0.01)	0.108
	MR-PRESSO (4 outliers)	−0.06 (−0.13, 0.01)	0.149
	Contamination mixture	−0.11 (−0.15, −0.08)	0.020
	MR-Mode	−0.10 (−0.21, 0.02)	0.130
	MR-Mix	−0.14 (−0.22, −0.06)	0.001
	GSMR (<10 SNPs)	NA	

*NA* not applicable given that the genetic instrument for the ADHD exposure included only 9 SNPs, GSMR is not a suitable method

**Table 4:** MR-CAUSE analysis displaying that the sharing model has a significantly worse fit than the causal model for the association between ADHD and the outcomes education, income, TDI, as well as intelligence, respectively

Model 1^a^	Model 2^a^	Δ ELPD^b^	s.e. Δ ELPD	***z***-score	***p***-value
**Education (SNPs=1430)**					
Null	Sharing	−6.9	2.2	−3.2	0.00072
Null	Causal	−13.0	4	−3.3	0.00041
Sharing	Causal	−6.4	1.8	−3.5	0.00023
**Income (SNPs=1430)**					
Null	Sharing	−3.1	1.5	−2.1	0.018
Null	Causal	−8.2	3.6	−2.3	0.011
Sharing	Causal	−5.1	2.1	−2.4	0.008
**TDI (SNPs=1430)**					
Null	Sharing	−5.2	2.2	−2.4	0.0092
Null	Causal	−10.0	4.1	−2.5	0.007
Sharing	Causal	−4.8	1.9	−2.5	0.0063
**Intelligence (SNPs=1430)**					
Null	Sharing	−0.91	0.77	−1.2	0.12
Null	Causal	−4.60	2.7	−1.7	0.043
Sharing	Causal	−3.70	1.9	−1.9	0.027

^a^Model 1 and Model 2 refer to the models being compared (null, sharing, or causal)

^b^Model fit is measured by Δ Expected Log Pointwise Posterior Density (ELPD); Negative values indicate that model 2 is a better fit

## Discussion

Previously, the independence and direction of the associations between a diagnosis of ADHD with different markers of socioeconomic and intelligence have been unclear. Our results based on a bidirectional two-sample MR-analysis design show that a genetic predisposition to socioeconomic position including attained educational level, household income, and TID has a direct and strong intelligence-independent association with ADHD. Genetically predicated intelligence per se was not independently associated with ADHD. Genetic liability to ADHD was only modestly inversely associated with SES measures and not clearly with lower intelligence.

Using the bidirectional MR design, our study provides a deeper understanding of previous observational findings [14–18, 29] by clarifying the directional strengths of the associations of ADHD with different aspects of SES as well as with intelligence. Our study also corroborates findings from a recent Mendelian randomization study [25] that reported a strong intelligence-independent association between genetic liability to higher education with lower odds of ADHD, and only a moderately strong association in the reverse direction. We found that the impact of several different aspects of socioeconomic factors on the diagnosis of ADHD was considerably stronger than the influence of an ADHD diagnosis on attained educational level, household income, and TID. The findings also indicate that intelligence is not a major cause or consequence of an ADHD diagnosis, associations suggested in previous observational analyses [4, 43]. It should be emphasized that we are not using the actual measures but genetic instruments for lifetime SES, intelligence, and ADHD with the sequential ordering of events estimated by the bi-directional approach rather than by a timed course of events for the actual exposures and outcomes.

One possible explanation for the fact that we see that SES-related factors are strongly linked to the receipt of an ADHD diagnosis may be that social vulnerability, lack of cultural, and economic capital or immaturity can be a basis for difficulty concentrating and sitting still, e.g., in a school environment. Lack of cultural capital, in the form of the parents’ level of education, can be manifested as shortcomings in language competence, for example. Parental SES is related to language development in multiple domains throughout both childhood and adulthood [44]. Children from lower SES homes show on average lower sustained levels of language and communicative skill than children from higher SES homes, differences beginning in infancy [44]. Low maternal education in general, single parenthood, and social welfare support are thought collectively to account for more than half of all ADHD medication prescriptions in Sweden [45], a country with a tax-supported health care system. In addition, children and young people from more deprived backgrounds in other areas Europe are also more likely to receive medication for ADHD [45, 46].

The resourceful family may also have greater opportunity to protect, stimulate, and train their children to become physically and mentally competent and thus avoid an ADHD diagnosis [47]. In addition to those possible mechanisms for low SES to lead to a diagnosis of ADHD, there are also non-causal possibilities for the association. A possible partial explanation might be that resourceful families, and individuals are reluctant to carry out the investigation leading to an ADHD diagnosis since a diagnosis limits rather than improves the individual’s opportunity for successful choices in life. This may involve a requirement for a medical certificate to obtain a driving license or disqualification from the possibility to obtain specific occupations. An additional aspect may be that an ADHD diagnosis fits poorly with the social practices in which a controlled temper is expected to accompany the physical and mental abilities that characterize a cultivated person [47]. A controlled body and mind with an ability for sustained attention is a classic aristocratic ideal with historical roots from antiquity [48–50].

By some measures, children of lower socioeconomic status score more than 2 years behind their more well-off peers on standardized language development tests by the time they enter school [51]. As a result, an individual from a lower status family may have difficulty following longer instructions, trouble getting started with tasks, and problems with attention in general. A reasonable theoretic interpretation is that these difficulties can lead to frustration and a range of behaviors that comprise ADHD.

Indirect support for the view that immaturity can affect the likelihood to being diagnosed with ADHD, is the fact that children in Norway born in October through December are at 50% higher risk of being diagnosed and treated for ADHD compared with children born the same year but in January through March [52]. Children who are almost a year younger tend also to appear more immature than their classmates, which influences both their academic and physical performance. The youngest children in a grade are often developmentally less mature and are more likely to behave more inattentively, impulsively, and hyperactively than their older classmates [53]. Furthermore, the association between SES with school readiness and maturity is well established and globally observed [54, 55]. Thus, for some ADHD cases, there conceivably could have been a tendency to confuse immaturity with ADHD, a possibility that can be supported by the fact that half of the people who were diagnosed with ADHD as children do not sustain the diagnosis in young adulthood [4].

Collectively, our findings of a strong 3–5 fold change in the odds of ADHD diagnosis per SD change in genetically predicated various dimensions of SES raise questions about whether the current criteria for diagnosis are sufficiently accurate and culturally generalizable tools to correctly identify ADHD, a descriptive diagnosis for which there is not yet any clinically valid biological marker. We definitely acknowledge that ADHD is regarded as a highly debilitating and costly disease [4], but our results suggest that refinement of the diagnosis may be warranted since there may exist subgroups with the diagnosis, especially in those with low SES.

In the Psychiatric Genomics Consortium GWAS, genetic markers for ADHD were identified either by an ADHD diagnosis or by having been prescribed ADHD medications, though without heterogeneity [9]. Therefore, our results can be generalized to both these categories. This is of interest since treatment with ADHD medication alone seems similarly effective in children from families with low and high socioeconomic status whereas combined medication and behavioral treatment showed a superior effect only in children from educated families [56]. Evidence of treatment effects in placebo-controlled randomized clinical trials with amphetamine-like compounds, average a 0.5–0.8 SD reduction in ADHD symptom scoring [4, 57]. A comparable treatment effect on similar measured modalities has also been observed in placebo-controlled trials in healthy young adult volunteers [58].

### Strengths and limitations

Using genetic data from large-scale GWASs and genetic consortia, the present study is the first to comprehensively and jointly investigate the causal effects of different aspects of SES and intelligence on the diagnosis of ADHD and vice versa. In addition, we disentangled the independent effect of socioeconomic factors from intelligence using a multivariable MR approach. The analyses were based on data from individuals of the European ancestry thereby limiting the potential for population stratification bias. However, this restriction limits the transferability of the present findings to populations of the non-European ancestries.

Our genetic instruments for social class were not strong, as measured by the explained variance. However, this potential limitation can be regarded of less importance given our two-sample design, a large number of SNPs in the SES instrument variables, and large sample sizes [59, 60]. In any case, any bias due to weak instruments discovered in non-overlapping cohorts will be in the direction of the null [59, 60], supporting our findings of the associations of socio-economic factors leading to a diagnosis of ADHD.

Another conceivable serious limitation of the present study is the possibility of unbalanced horizontal pleiotropy from genetic variants acting through several different biological pathways. Thus, an alternative underlying causal explanations for the SES impact of on the risk of ADHD diagnosis might be factors linked to low SES such as parental mental health, substance abuse, and maternal smoking during pregnancy [14]. Carefully conducted studies have nevertheless not shown a major impact of maternal smoking or substance abuse on ADHD diagnosis [61, 62], and our findings in several different sensitivity analyses do not indicate major pleiotropic or genetically correlated influences that could explain the impact of genetic liabilities of SES on ADHD.

Non-genetic familial influences could not directly be assessed in the present MR study and for obvious reasons, some SES genetic liability markers in our study (household income, car ownership, educational level, and unemployment) pertain to the parent’s circumstances when a child receives an ADHD diagnosis. The genetic liability of low or high SES still pertains to the child. Nonetheless, our design precludes the evaluation of timing of exposure effects and induction periods on the occurrence of the outcomes. The strength of the associations from the analyses in different directions are not directly comparable, but the interpretation of the overall pattern was straightforward. Since identification of socioeconomic patterns with specific ADHD subtypes was not in the scope of our study, we suggest refined evaluation of different disease development pathways between SES and ADHD.

## Conclusions

We conclude that there is a strong direct impact of genetically predicted household income, educational attainment, and social deprivation index on the risk of an ADHD diagnosis. Lower intelligence seemed not to be a strong independent cause or a consequence of ADHD. The associated effect of genetic liability of ADHD on genetically predicted socioeconomic markers were modest.

## Supplementary Information

**Additional file 1: Table S1.** Data sources for the genetic instruments.

## Data Availability

All the data supporting the conclusions of this article are included within the article and its supplementary file.

## Abbreviations

ADHD: Attention-deficit/hyperactivity disorder
SNP: Single-nucleotide polymorphism
GWAS: Genome-wide association study
SES: Socioeconomic status
MR: Mendelian randomization
TID: Townsend index of deprivation
ICD: International Classification of Diseases
IVW: Inverse-variance weighted
ORs: Odds ratios
MR-PRESSO: Pleiotropy RESidual Sum and Outlier
GSMR: Generalised Summary-data-based Mendelian Randomisation
ELPD: Expected Log Pointwise Posterior Density
