# Supplementary material for: The impact and causal directions for the associations between diagnosis of ADHD, socioeconomic status, and intelligence by use of a bi-directional two-sample Mendelian randomization design
Source: BMC Med. 2022 Apr 11;20:106. doi: 10.1186/s12916-022-02314-3 (PMC8996513; doi:10.1186/s12916-022-02314-3)
Supplement: Supplementary file 1 — Additional file 1: Table S1. Data sources for the genetic instruments. [file 12916_2022_2314_MOESM1_ESM.docx]

**Table S1.** Data sources for the genetic instruments

| **Trait** | **PMID** | **MR_base_ID** | **Case** | **Control** | **SNPs** | **Palindromic** |
| --- | --- | --- | --- | --- | --- | --- |
| Intelligence | 29942086 | ebi-a-GCST006250 | - | 269867 | 135 | 19 |
| Education | 30038396 | ieu-a-1239 | - | 766345 | 260 | 40 |
| Household income | Not published (MRC-IEU, Ben Elsworth) | ukb-b-7408 | - | 397751 | 44 | 3 |
| Townsend deprivation index | Not published (MRC-IEU, Ben Elsworth) | ukb-b-10011 | - | 462464 | 18 | 0 |
| ADHD | 30478444 | ieu-a-1183 | 35191 | 55374 | 11 | 1 |

**References and data sources:**

Intelligence: Jeanne E Savage et al. Genome-wide association meta-analysis in 269,867 individuals identifies new genetic and functional links to intelligence. Nat Genet 2018; doi: 10.1038/s41588-018-0152-6; MRBASE <https://www.mrbase.org/> (2018)

Education: James J Lee et al. Gene discovery and polygenic prediction from a genome-wide association study of educational attainment in 1.1 million individuals. Nat Genet 2018; doi: 10.1038/s41588-018-0147-3; MRBASE <https://www.mrbase.org/> (2018)

Household income: Not published but data can be accessed at MRBASE <https://www.mrbase.org/> (2021)

Townsend deprivation index: Not published but data can be accessed at MRBASE <https://www.mrbase.org/> (2021)

ADHD: Ditte Demontis et al. Discovery of the first genome-wide significant risk loci for attention deficit/hyperactivity disorder. Nat Genet 2019; doi: 10.1038/s41588-018-0269-7; MRBASE <https://www.mrbase.org/> (2019)
